# Free-ranging dogs show age related plasticity in their ability to follow human pointing

**DOI:** 10.1371/journal.pone.0180643

**Published:** 2017-07-17

**Authors:** Debottam Bhattacharjee, Nikhil Dev N., Shreya Gupta, Shubhra Sau, Rohan Sarkar, Arpita Biswas, Arunita Banerjee, Daisy Babu, Diksha Mehta, Anindita Bhadra

**Affiliations:** 1 Department of Biological Sciences, Indian Institute of Science Education and Research Kolkata, Nadia, West Bengal, India; 2 Indian Institute of Science Education and Research Thiruvannathapuram, Kerala, India; 3 Indian Institute of Science Education and Research Bhopal, Madhya Pradesh, India; 4 Shivaji College, University of Delhi, Delhi, India; University of Portsmouth, UNITED KINGDOM

## Abstract

Differences in pet dogs’ and captive wolves’ ability to follow human communicative intents have led to the proposition of several hypotheses regarding the possession and development of social cognitive skills in dogs. It is possible that the social cognitive abilities of pet dogs are induced by indirect conditioning through living with humans, and studying free-ranging dogs can provide deeper insights into differentiating between innate abilities and conditioning in dogs. Free-ranging dogs are mostly scavengers, indirectly depending on humans for their sustenance. Humans can act both as food providers and as threats to these dogs, and thus understanding human gestures can be a survival need for the free-ranging dogs. We tested the responsiveness of such dogs in urban areas toward simple human pointing cues using dynamic proximal points. Our experiment showed that pups readily follow proximal pointing and exhibit weaker avoidance to humans, but stop doing so at the later stages of development. While juveniles showed frequent and prolonged gaze alternations, only adults adjusted their behaviour based on the reliability of the human experimenter after being rewarded. Thus free-ranging dogs show a tendency to respond to human pointing gestures, with a certain level of behavioural plasticity that allows learning from ontogenic experience.

## Introduction

Humans have domesticated several animal species over thousands of years, beginning with the dog [1]. Generations of close association between humans and the various domesticated species has led to the development of certain degrees of communication and attachment with humans in these species. Several such species including goats, pigs, ferrets, horses, cats etc. have been shown to respond to human communicative intents [2–6]. However, probably due to high degree of bonding present between dogs and humans, the social cognition of dogs (*Canis lupus familiaris*) and their ability to interact with humans have made them one of the most extensively studied species in the recent past [7]. Dogs are capable of interacting and establishing social bonds with humans, which are similar to human caregiver-infant relationships [8]. It has even been suggested that dogs “need” to bond with humans, and the presence of a human can help reduce stress in dogs [9]. Human interventions like positive reinforcement and affiliative interactions are expected to influence dog behaviour positively, thereby being important factors in the training of dogs by their handlers [10,11].

Dogs are highly responsive to human-provided social cues and perform successfully in the “object choice tasks”, as opposed to primates including chimpanzees that perform poorly [12]. Several studies have explored the extent of social cognition in dogs, and their ability to comprehend human communicative gestures. They have been shown to locate hidden food rewards by following different pointing cues from humans [13–16]. Dogs not only are able to follow human pointing gestures, but are also capable of using the body postures of humans to understand different referential gestures made by humans [17]. Interestingly, unlike the pet dogs, their closest relatives, the gray wolves (*Canis lupus lupus*), differ in utilizing human pointing gestures at different stages of their development because of less socialization and delayed emergence of such behaviour [18]. Thus, in spite of their common ancestry and high genetic similarity, dogs and wolves possess somewhat different socio-cognitive abilities.

The origin and probable modification of socio-cognitive skills in dogs have been a highly debated topic leading to different hypotheses mentioning the importance of both domestication and ontogeny [19,20]. Studies with socialized wolves and shelter dogs have established the role of the environment, different levels of human socialization and ontogenic experiences in shaping these skills [21]. Some authors have suggested co-evolution with humans [22], and the presence of a theory of mind in dogs [19] to be key factors for their high socio-cognitive abilities. A somewhat conservative explanation suggests that sensitivity to the behaviours of both humans and conspecifics is likely to be advantageous for scavengers living in social groups and thus, a predisposition in the dogs to attend to human actions can allow for rapid early learning of the association between certain gestures and food availability [23]. Studying socio-cognitive abilities of free-ranging populations of dogs can help us understand if behavioural ecology has indeed shaped this ability in dogs through the process of evolution.

Free-ranging dogs comprise nearly 80% of the world’s dog population, and they are largely free-breeding populations that are not under direct human supervision [24–28]. They not only represent a more genetically diverse group, but are also closer to the present day wolves genetically, than the pets which have been produced by generations of artificial selection [29,30]. Free-ranging dog populations across the world experience differing levels of human interactions, and being close to humans but not being reared by them, represent a unique condition for carrying out tests of socio-cognitive abilities. In India, free-ranging dogs have existed as continuous populations for centuries [31] and are a ubiquitous presence in all human habitations, from metropolitan areas to remote villages [32]. They are mostly scavengers, relying primarily on human-generated waste for sustenance, and on the other hand, also receive negative impact from humans. A recent study has shown that 63% of total mortality of free-ranging dogs in India is influenced by humans [33]. Free-ranging dogs are often considered as a menace because they scatter garbage, defecate by the streets and disturb people by their nocturnal barking; they are also sources of zoonotic diseases like rabies [34–36]. Thus, unlike pets, the free-ranging dog-human relationship is highly complex and needs proper understanding. Several studies have pointed out the need for testing the socio-cognitive abilities of free-ranging dogs, which is greatly lacking and under-represented currently [37,38]. This is the very first time that cognitive experiments on free-ranging dogs have been performed, pertaining to the use of simple human pointing gestures. We have considered three distinct developmental phases of these dogs—pup, juvenile and adult to understand this complex dog-human relationship in a greater detail from the perspective of ontogeny as these phases differ in their levels of human socialization and impact [33].

Lack of cognitive studies on free-ranging dogs, especially using the human communicative intents, have led us to start testing with one of the simplest form of pointing gestures, the dynamic proximal pointing. We have complemented the success rates using behavioural data. For example, instead of discarding the subjects that did not approach even after a successful familiarization phase, we included them in the analysis and calculated success rates for a better understanding of their behavioural profiles. We have specifically emphasized on gaze alternation behaviour which is considered to be a requesting behaviour for some species in order to get the information of a hidden food reward [39,40]. Studies have shown that dogs even try to use misleading cues given by humans either by approaching an empty container after having noticed or sniffed a baited container [41]; or by choosing a plate with smaller quantity of food [42]. A study by Takaoka et al. [43] has shown dogs’ behavioural modifications of using cues based on the inference of reliability of a human experimenter. We have performed three consecutive trials to check whether dogs can adjust their response to human pointing based on reliability of the human experimenter.

## Materials and methods

### Subjects and study sites

The study was carried out in and around the IISER Kolkata campus at Mohanpur (22°56’49”N and 88°32’4”E) and Kalyani (22°58’30”N, 88°26’04”E), West Bengal, India. The subjects were 83 adult free-ranging dogs, 58 juveniles and 68 pups (S1 and S2 Tables), tested individually at locations such as residential areas, market places, railway stations and areas adjacent to highways. The experiments were carried out at random times between 0700 hours and 1800 hours from June 2015 to July 2016, in order to cover all the developmental phases of dogs. Two experimenters were involved in the experiment at any point of time, and have been designated as E1 (experimenter 1) and E2 (experimenter 2), irrespective of their identity. The experimenters walked on streets to locate solitary adult dogs and juveniles randomly for experimental trials. Pups were usually lured away from the group for the experiment. Sexes of adult dogs were confirmed by looking at their genitals. Pups and juveniles need to be inspected closely for sexing, and in order to avoid handling the subjects, we did not record their sexes for this study. To rule out any possibility of resampling subjects, we tested dogs from different locations on different days and photographed each individual.

### Experimental procedure

Three opaque plastic bowls (Volume = 500 ml) and card-board pieces (as covers) were used for the experiments with adults and juveniles. Similarly, smaller plastic bowls (Volume = 100 ml) and card-board pieces were used for pups. We provided a piece of raw chicken as the hidden food reward. A familiarization phase, followed by three consecutive trials were conducted for both the test and control conditions. The experimenter (E2) bent down slightly while pointing, using the index finger. This was similar to the dynamic proximal pointing method reported by Miklosi and Soproni’ 2006 [15], but kneeling had to be avoided due to two reasons–free-ranging dogs often showed alarm when someone kneeled before them during the pilot experiments and did not approach most of the time (S1 Text); the experiments were conducted in the dogs’ natural habitats, which were not always convenient locations for kneeling down. The protocol was consistent while testing all three categories of dogs.

#### Familiarization

Experimenter 1 (E1) attracted the attention of an individual dog, placed a piece of raw chicken in a plastic bowl and covered it with a card-board piece. This process was carried out in order to familiarize the dog with the set up. The dog was allowed to approach the bowl and interact with it for a total period of 30s. Only the dogs that successfully completed the familiarization phase (i.e., approached the bowl and obtained the piece of meat) were used for either the test or the control experiment. E2 did not witness the familiarization phase.

#### Test

The test phase was carried out immediately after the familiarization phase, and consisted of three consecutive trials. E1 placed a food reward randomly in one of two fresh bowls out of the range of vision of the subject and E2, thereby ensuring that the experiment was double-blind. To control for the smell of chicken in the baited bowl, the other bowl was sham baited by rubbing a piece of chicken inside it. Both bowls were then covered by E1 using pieces of cardboard. E1 left the set-up area after handing over the bowls to E2, who then placed both the bowls on the ground equidistant (1.2–1.5 m) from the dog. E2 tried to establish eye-contact with the dog and provided the pointing cue on catching the dog’s attention. Most free-ranging dogs have no specific names like pet dogs, but people typically call out to them using communicative signals or words, which were used by E2 during the experiment (S1 Video). When the focal dog looked at E2, he/she pointed randomly at one of the bowls, bending towards the bowl in such a way that the distance between the index finger of the extended arm and the bowl was approximately 10–15 cm. (S1 Fig; S2 Video). E2 looked at the dog throughout the trial while providing the pointing cue. Since the subjects were tested without the help of a leash and were thus free to move around, E2 placed the bowls such that the distance between the subject and the bowls were approximately the same in every trial. The process was video recorded for 30 seconds or till the dog made its choice by approaching the set-up, whichever was earlier. Choice was defined as the dog approaching any of the bowls (irrespective of pointing cue) and removing the cover to inspect the bowl. If the dog found the reward, it was allowed to take it. Three trials were carried out with 5–10 seconds intervals in between trials. After the dog made its choice, or 30 seconds, whichever was earlier, the contents of both containers were revealed to the dog.

#### Control

The control trials were carried out with a set of individuals not used for the test trials and followed immediately after the familiarization phase, exactly like the test trials. Here, E2 did not provide any cue, stood in a neutral posture and looked straight ahead without making eye contact with the focal dog (S2 Fig). The procedure was otherwise the same as the test trials. Controls were done to rule out further possibilities of olfactory cues and effect of motion or orientation response hypothesis [44].

### Data analysis and statistics

All the videos were coded by a single experimenter for task performance and associated behaviours, which were then used for further analysis. Only trial 1 data were used for analysis except in the case of reliability scoring to ensure no effect of bias due to previous trials. We compared pups, juveniles and adults to understand the effects, if any, of ontogeny on the free-ranging dogs’ point-following behaviour. Post-hoc pairwise comparison with Bonferroni correction was done wherever required. We used Shapiro-Wilk tests to check for normality of our data and found them not normally distributed, thus we performed non-parametric tests.

We compared the following parameters between pups, juveniles and adults: (i) proportion of individuals approaching and not approaching the task using 2X2 Contingency chi-square test; (ii) out of the dogs that approached, we compared the proportion of dogs that actually followed pointing cues, using Chi-square test; (iii) latency to approach the task using Kruskal-Wallis test; (iv) survival analysis for representation of individuals approaching and not approaching and latency to approach; (v) occurrence of gaze alternation using Chi-square test; (vi) frequency and duration of gaze alternation behaviour using Kruskal-Wallis tests. For post-hoc pairwise comparisons in case of latency to approach the set-up, frequency, and duration of gaze alternation, we used Mann-Whitney U test. We compared the test and control conditions for pups, juveniles and adults separately.

In order to understand whether dogs adjust their point following behaviour based on the reliability of the human experimenter, two parameters were considered–‘positive reinforcement’ and ‘lack of reinforcement’. Positive reinforcement was calculated by the proportion of individuals who followed pointing in a trial and obtained a reward in the same trial (+ + condition) and lack of positive reinforcement was estimated by the proportion of individuals who followed pointing but did not receive a reward in the trial (+—condition). We estimated the proportion of individuals that followed pointing in the consecutive trial after positive reinforcement (+ + /+ condition) and those that did not follow pointing after lack of positive reinforcement (+—/- condition) as measures of behavioural adjustments of dogs based on reliability of the human experimenter. We used data from two sets of consecutive trials (trials 1 and 2, trials 2 and 3) for these calculations.

We used Generalized Linear Models (GLM) to check the relationship between following and not following pointing cue with the frequency and duration of gaze alternation for pups, juveniles and adults. We performed a 2X2 Contingency chi-square test to investigate any effect of age on the point-following behaviour of pups. A second coder naïve to the purpose of the study coded 20% of the trials to check inter-rater reliability. It was perfect for point following behaviour (cohen’s kappa = 1.00) and almost perfect for gaze alternation behaviour (cohen’s kappa = 0.90) [45]. The alpha level was 0.05 throughout the analysis. GLMs were performed using “lme4” package of R version 3.0.2 [46]. Along with R, other statistical analyses were performed using StatistiXL version 1.11.0.0.

## Results

### Test

**Fig 1:**
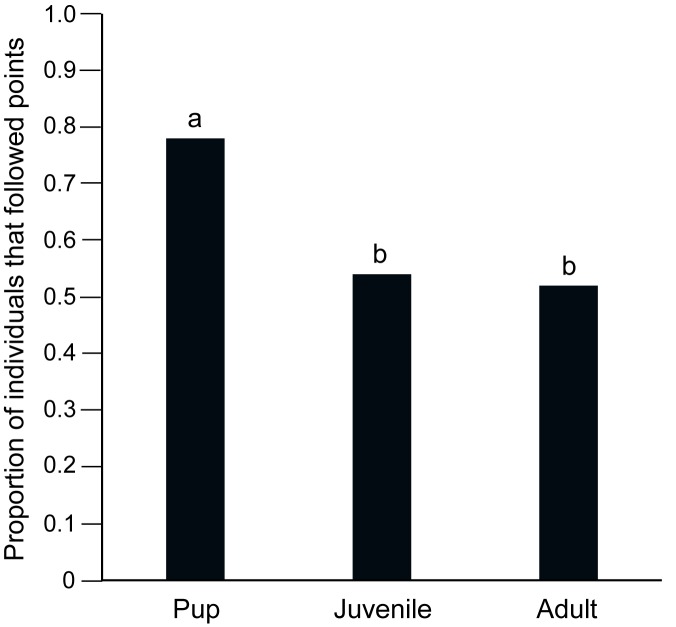
Bar graph showing the proportion of pups, juveniles and adults that followed pointing cue. Pups followed pointing cues significantly higher than juveniles (contingency χ^2^, p = 0.03) and adults (contingency χ^2^, p = 0.02). “a” and “b” indicate significant differences among the categories.

89% of the pups, 43% of the juveniles and 69% of the adults approached either of the two bowls. Comparison between “approach” and “no approach” categories among the pups, juveniles and adults showed significant variation (Contingency χ^2^ test: χ^2^ = 15.752, df = 2, p < 0.001), all three categories of dogs showing significantly different levels of approach from each other. Contingency chi-square tests revealed that pups showed higher approach levels than both juveniles (χ^2^ = 15.654, df = 1, p < 0.001) and adults (χ^2^ = 4.477, df = 1, p = 0.03), while juveniles showed lesser approach than adults (χ^2^ = 4.761, df = 1, p = 0.03). Of the individuals that approached the bowls, 78% of the pups, 54% of the juveniles and 52% of the adults actually followed the pointing cue. Significantly higher proportion of pups followed pointing, as compared to juveniles (Goodness of fit: χ^2^ = 4.364, df = 1, p = 0.03) and adults (Goodness of fit: χ^2^ = 5.200, df = 1, p = 0.02), while the proportion of juveniles and adults that followed pointing did not differ significantly (Goodness of fit: χ^2^ = 0.038, df = 1, p = 0.85; Fig 1). The mean latency times to approach were 3.12 sec, 3.92 sec and 5.78 sec for pups, juveniles and adults respectively and were not different for the three categories (Kruskal-Wallis test: F = 5.588, df = 2, 68, p = 0.061).

We used survival analysis (S2 Text) to show proportion of individuals approaching and not approaching the set-up with their latencies. We have considered approaching the set-up as events and thus probability of not approaching was considered as survival probability. A Cox proportionate hazard model was constructed for testing pups, juveniles and adults. According to the model, we found a significant difference between pups (p = 0.005) and adults, but adults and juveniles (p = 0.09) remained the same as mentioned in Table 1.

**Table 1:** Result of Cox proportionate hazard test for comparing the approach of adults, juveniles and pups with their latencies.

Covariate	Hazard ratio	Lower CL	Upper CL	p
**Juveniles**	0.57	0.29	1.10	0.09
**Pup**	2.08	1.24	3.49	0.005

Hazard ratio depicts how many times the probability of approaching or not approaching the set-up of the covariates are compared to the adults. The lower and upper CL (confidence limit) gives the confidence interval of the hazard ratio.

**Fig 2:**
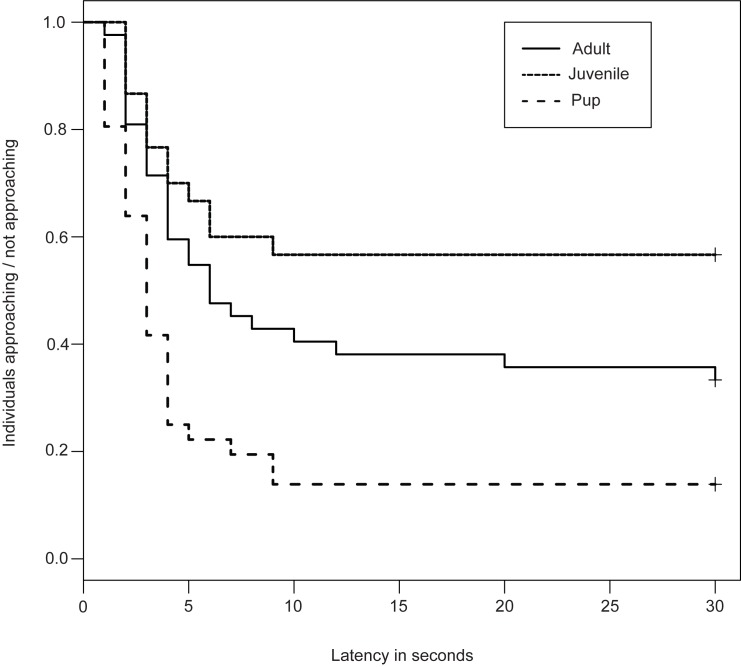
Survival model showing proportion of individuals approaching the set-up and their latencies. Dotted line, solid line and dashed line indicates the survival curves of juveniles, adults and pups respectively. Latency to approach the set-up is plotted in Y-axis and proportion of approach in X-axis.

Survival curves of pups, juveniles and adults are given in Fig 2.

**Fig 3:**
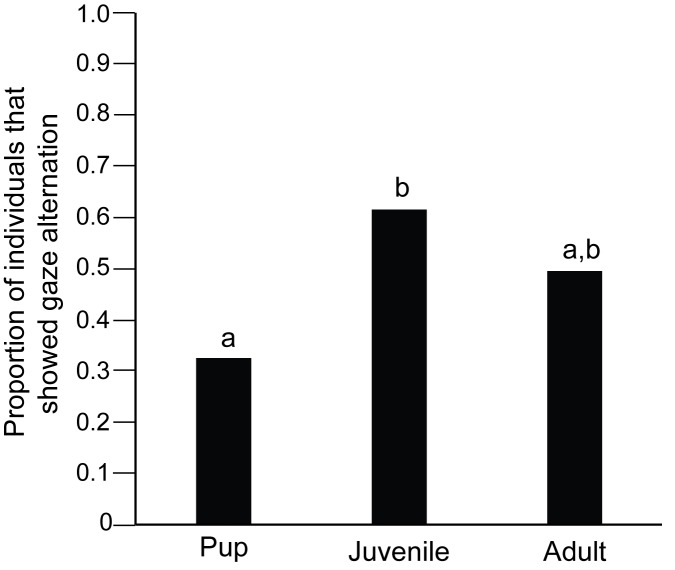
Bar graph showing the proportion of pups, juveniles and adults that showed gaze alternation behaviour. Occurrence of gaze alternation was significantly higher in juveniles than pups (Goodness of fit, p = 0.01). “a” and “b” indicate significant differences between the categories.

**Fig 4:**
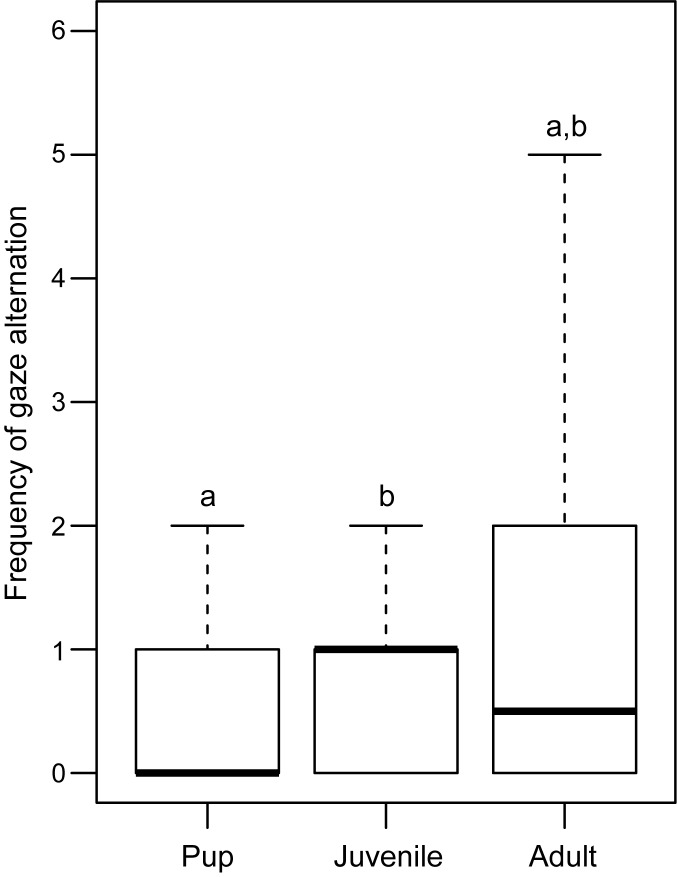
Box and whiskers plot showing the frequency of gaze alternation shown by pups, juveniles and adults. Frequency of gaze alternation was significantly higher in juveniles than pups (Mann Whitney U test, p = 0.029). Boxes represent interquartile range, horizontal bars within boxes indicate median values, and whiskers represent the upper range of the data. “a” and “b” indicate significant differences among the categories.

**Fig 5:**
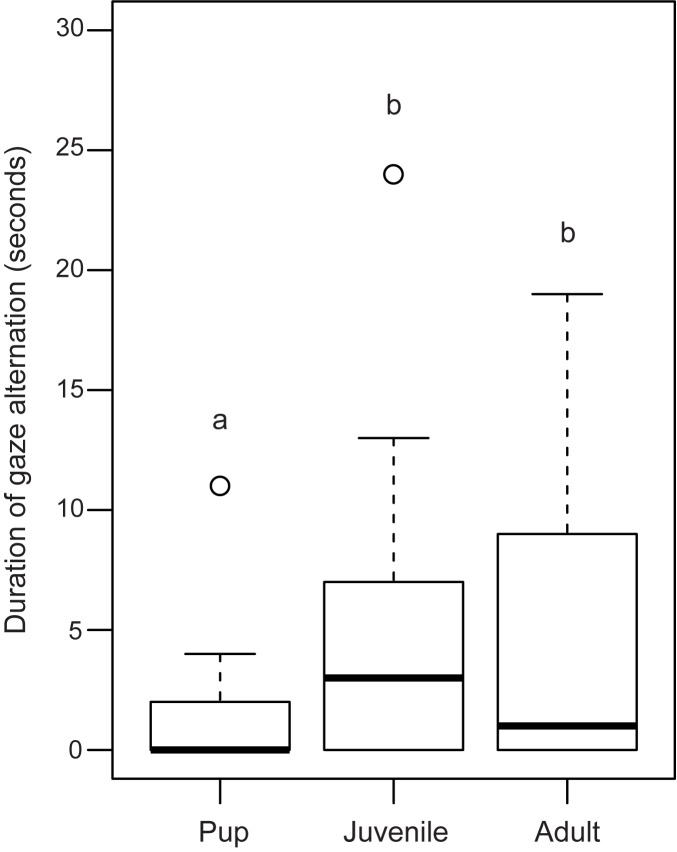
Box and whiskers plot showing duration of gaze alternation behaviour by pups, juveniles and adults. Duration of gaze alternation was significantly less prolonged in pups than in juveniles (Mann Whitney U test, p = 0.003) and adults (Mann Whitney U test, p = 0.04). Boxes represent interquartile range, horizontal bars within boxes indicate median values, and whiskers represent the upper range of the data. “a” and “b” indicate significant differences among the categories.

33% of the pups, 62% of the juveniles and 50% of the adults tested in Trial 1 (test) showed gaze alternation behaviour. The proportion of juveniles showing gaze alternation was higher than that of pups (Goodness of fit: χ^2^ = 8.853, df = 1, p = 0.003), but did not differ significantly from the adults (Goodness of fit: χ^2^ = 1.286, df = 1, p = 0.25). The proportions also did not differ significantly between adults and pups (Goodness of fit: χ^2^ = 3.48, df = 1, p = 0.06; Fig 3). There was significant variation in the frequency of gaze alternation among the three categories of individuals (Kruskal-Wallis test: F = 3.618, df = 2, 103, p = 0.032). Post hoc analysis revealed significant difference in the frequency of gaze alternation between pups and juveniles (Mann Whitney U test: U = 687.000, df = 36, 29; p = 0.029). Adults showed no significant difference with either pups (Mann Whitney U test: U = 953.000, df = 36, 42; p = 0.05) or juveniles (Mann Whitney U test: U = 624.50, df = 29, 42; p = 0.85; Fig 4). There was significant variation in the duration of gaze alternation behaviour among the pups, juveniles and adults (Kruskal-Wallis test: χ^2^ = 10.408, df = 2, p = 0.005), with pups showing less prolonged gaze alternation than juveniles (Mann Whitney U test: U = 748.00; df = 36,29; p = 0.003) as well as adults (Mann Whitney U test: U = 973.00; df = 36,42; p = 0.03), while juveniles and adults did not differ significantly (Mann Whitney U test: U = 642.00; df = 29,42; p = 0.70; Fig 5).

Adults followed the pointing cue in significantly higher rates in trials 2 and 3 when positive reinforcement occurred in trials 1 and 2 respectively (14 out of 16 instances; Goodness of fit: χ^2^ = 57.760, df = 1, p < 0.0001). In addition to this, adults did not follow pointing cue in trials 2 and 3 when lack of positive reinforcement occurred in trials 1 and 2 respectively (4 out of 6 instances; Goodness of fit: χ^2^ = 11.560, df = 1, p = 0.001). Thus adults seem to be quite flexible in point-following behaviour, adjusting their decision based on the reliability of the human experimenter. Juveniles did not show any difference in terms of behavioural adjustment as they showed a 50–50 response when they obtained food reward (5 out of 10 instances; Goodness of fit: χ^2^ = 0.000, df = 1, p = 1). They showed a marginal tendency to follow the cue less in case of lack of positive reinforcement (3 out of 5 instances; Goodness of fit: χ^2^ = 4.000, df = 1, p = 0.05). Pups showed a response in trial 2 and trial 3 exactly like the juveniles when they followed and obtained food in trial 1 and trial 2 respectively (7 out of 14 instances; Goodness of fit: χ^2^ = 0.000, df = 1, p = 1). Interestingly, pups relied on the experimenter and followed pointing cue even after lack of positive reinforcement (11 out of 17 instances; Goodness of fit: χ^2^ = 9.000, df = 1, p = 0.003), suggesting their inability to adjust point-following behaviour like adults and an innate tendency to socialize with humans. GLM analysis revealed only the frequency of gaze alternation to be a significant predictor for point following in pups (GLM: Z = -3.014, p = 0.002; S3 Text). GLM analysis for juveniles and adults were not significant for any of the variables. We found no effect of age (4 to 8 weeks) on the tendency to follow pointing in pups (Contingency χ^2^ test: χ^2^ = 4.011, df = 4, p = 0.405; S3 Fig).

#### Control

We did not find any difference between the rates of “approach” and “no approach” among pups, juveniles and adults (Contingency χ^2^ test: χ^2^ = 1.350, df = 2, p = 0.5). The rates of obtaining a food reward and not obtaining the same were not different (Contingency χ^2^ test: χ^2^ = 1.347, df = 2, p = 0.5). Within category comparison revealed that both pups and juveniles failed at significantly higher rates to get the food reward in the no cue condition (Pup–Goodness of fit: χ^2^ = 23.040, df = 1, p < 0.001; Juvenile - χ^2^ = 40.960, df = 1, p < 0.001), but adults did not (χ^2^ = 1.960, df = 1, p = 0.16). We did not find any difference in the occurrence (Goodness of fit: χ^2^ = 4.850, df = 2, p = 0.08), frequency (Kruskal-Wallis test: χ^2^ = 5.445, df = 2, p = 0.07) and duration of gaze alternations (Kruskal-Wallis test: χ^2^ = 4.711, df = 2, p = 0.09) among the pups, juveniles and adults.

#### Test-control comparison

**Fig 6:**
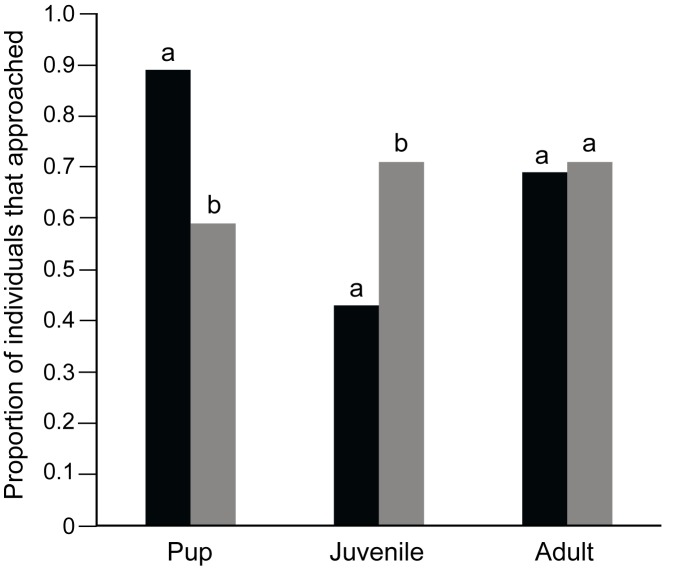
Bar graphs showing the proportion of pups, juveniles and adults that approached the task in test and control conditions. Black bars indicate test conditions and grey bars indicate control conditions. Pups showed significantly higher approach rates in test condition than control (Goodness of fit, p = 0.01) and juveniles showed higher approach rates in control condition than test (Goodness of fit, p = 0.009). “a” and “b” indicate significant differences within the categories.

Pups approached significantly more in the test condition than the control (Goodness of fit: χ^2^ = 6.081, df = 1, p = 0.01), i.e, for pups, the tendency to approach was higher when pointing cue was provided. Interestingly, the approach rate of juveniles was much lower in the presence of the cue than in the absence of it (Goodness of fit: χ^2^ = 6.877, df = 1, p = 0.009). For the adults, the response levels were unaffected by the presence or absence of pointing cues (Goodness of fit: χ^2^ = 0.029, df = 1, p = 0.86; Fig 6).

## Discussion

Our experiment revealed interesting differences in the tendencies of free-ranging dogs to follow simple human pointing gestures at different stages of their development, thereby suggesting a role of ontogeny in the development of social cognition in these dogs. In our study, pups were most responsive to the task, and also showed the highest ability to follow dynamic proximal pointing. The relatively lower response of pups in the control condition further strengthened the validity of this conclusion. Juveniles acted in an interesting way by responding to the task more in the control condition than the test, and failing to follow the point in most cases. Adults showed similar response levels in the test and control conditions, suggesting that their tendency to respond to the task did not depend on the presence of the cue. Though pups followed pointing more than juveniles, only the adults showed adjustment of the point-following behaviour, based on their immediate experience. When an adult dog followed the pointing cue and obtained food reward, it had a higher chance of following the cue in the next trial; suggesting a level of flexibility in their reliance on humans. Juveniles showed a marginal tendency to avoid non-reliable humans more, while pups showed a higher tendency to rely even on non-reliable humans, suggesting a weaker avoidance of humans and inability to adjust the response to pointing based on an immediate experience of interaction with humans.

Several studies with pet dogs have shown that adult individuals are very sensitive and can use human communicative intents positively [47,48]. While most studies on adults show similar results, there is some disagreement regarding the ability of pups to follow human communicative gestures. A study by Dorey et al. (2010) showed that pups younger than 21 weeks had little ability to follow human pointing gestures, thereby suggesting a crucial role of ontogeny in the development of social cognition in dogs [20]. However, Gasci et al (2009) showed that 2 months old individuals performed at the same level as adults [49]. A recent study with shelter dogs again established the role of ontogeny and need of human socialization in order to dogs’ understanding of referential intents [21]. In our study, the pups were 4–8 weeks old, and thus in the pre-weaning stage of development, still in the care and protection of their mothers, with very limited exposure to humans [50]. The juveniles, on the other hand, were beyond the weaning stage [50–52], and had high levels of exposure to humans [33], comparable to that of the adults. A recent study compared the performance of pet and shelter dog pups in the age range of 20–24 weeks and concluded that pups’ ability to follow human pointing gestures was positively correlated with their experience with humans [53]. However, in this case, experience with humans is typically positive, whereas for free-ranging dog juveniles’ (3–7 months old), experience with humans can be both positive and negative [33,54].We did not see any effect of age (4–8 weeks) in the pups’ performance, but the drastic shift in the response level, tendency to follow pointing and gaze alternation behaviour in the juveniles, together with the positive effect of rewards on reliability in the adults clearly suggests behavioural plasticity for point-following in the free-ranging dogs. The varying responses to point-following behaviour could also be addressed by avoidance behaviour in these dogs incorporating the real effect of dog-human interaction. Earlier research suggests that young dogs have a critical period of socialization when they readily accept new people; followed by a marked period of fear, where unknown humans are intensively avoided [55,56]. In our experiment, adults could have gained ample experience with humans to recognize reliable and friendly people, but the juveniles might have experienced negative encounters that kept them away from the human experimenter. Juveniles approached the containers and the experimenter less frequently, when pointing cue was provided; this is again a good indicator of avoidance behaviour that strengthens this idea further.

In the current experimental design with dynamic proximal pointing, differences found among age groups could be due to different point-following capacity, or because of varying levels of fear of humans and thus intertwined and difficult to distinguish. In cognitive ethology, following pointing cues has become a standard testing paradigm because it may reflect the cognitive capacity of the subject understanding the communicative intention of the signaller; and additionally the capacity of understanding referential signals. Since proximal pointing is something where the distance between the target and the pointing finger is very short, it sometimes plays the role of a ‘marker’ or ‘beacon’. Thus, a positive response of the subject cannot be disentangled easily between a simple approach and directional signal or real pointing. While it is important to disentangle and establish the possible factors using complex gestures like momentary distal pointing, it is also necessary to figure out the responses of these dogs pertaining to the simplest form of pointing because of non-availability of earlier studies. Moreover, humans interact with dogs mostly using hands; thus approaching close to a human hand depicts attraction and not approaching the same as avoidance. In the streets, people beckon, feed and scare away dogs using their hands. Hence, dogs’ use of human proximal pointing is valid for both positive and negative intentions of humans, and further experiments need to be conducted to understand whether free-ranging dogs respond differently to different gestures of the human hand.

In India, free-ranging dogs constantly encounter humans in their daily lives, and humans are responsible for a large proportion of mortality in these dogs during the juvenile phase of life [33]. We suspect that the negative experiences with humans, such as beating, harassment or witnessing the death of pups, etc. make juveniles wary and perhaps afraid of humans, leading to a drastic reduction in reliability. Dogs are known not to rely on unreliable humans and they have the ability to adjust their behaviour accordingly [43,57]. In our study, the reliability on humans by the adults increased when they received a reward on following the pointing gesture, emphasizing the effect of experience in shaping the behaviour in free-ranging dogs. Studies have also shown differential behaviour of pups of free-ranging dogs than adults in terms of food preference and choice, once again suggesting a role of learning with experience [58]. It is interesting to note that similar effects of experience with humans have been shown in domestic pigs, in the context of an object choice task [59]. Pigs, like dogs, share a long history of domestication, and indeed are the earliest domesticated animals after dogs [60], and these concurrent observations provide insights into how humans influence the behavioural repertoire of animals that live in close association with them.

Gaze alternation behaviour is considered to be a requesting behaviour and an important means of social communication, which is shown by dogs (and also human infants) when they are faced with an unsolvable task and seek help from humans [39,61]. In our study, pups rarely altered their gaze whereas, juveniles and adults showed both higher frequency and duration of gaze alternation. Pups, on the other hand, mostly chose to follow the point. However, it is important to note that though the juveniles and adults showed lack of interest in humans in terms of point-following, they nevertheless had a tendency to communicate with humans through gaze alternation. Prolonged gazing can be attributed to both less interest in the hidden food and fear to approach the experimenter. Since we tested only those dogs that responded during the familiarization phase, lack of interest in the food is a less likely option. Hence the dogs’ dilemma to approach the bowls can be attributed to fear of the experimenter, which is high in juveniles and adults, and conspicuously missing in the pups. We speculate whether the gaze alternation behaviour could have served to assess human intention in the ancestral dogs, rather than seek help, and has been co-opted for communicating with humans through the process of taming. While the predisposition towards socializing with humans could have aided in the domestication process of dogs [12], the plasticity of this behaviour, enabling learning from ontogenic experience is crucial in helping dogs to adapt to life in a human-dominated landscape with ample situations of conflict between the two species.

### Ethics

No dogs were harmed or disturbed during this experiment. All experiments were performed in the natural habitats of the dogs, following a protocol approved by the IISER Kolkata animal ethics committee. All meat used in the experiment was fresh and fit for human consumption.

## Supporting information

S1 Table Sample sizes used for the three age categories in the experiment for test and control conditions. (PDF)

S2 Table Age wise sample size of pups used for test and control experiments. (PDF)

S1 Text Why kneel down posture was not used? (PDF)

S2 Text Cox proportional hazards test. (PDF)

S3 Text Effect of frequency of gaze alternation on pup’s point-following behaviour. (PDF)

S1 Video Practice of calling out free-ranging dogs in India. (MP4)

S2 Video Pointing gesture using an adult individual. (MP4)

S1 Fig Experimenter 2 pointing randomly at a bowl with one of the adult individuals. (PDF)

S2 Fig Experimenter 2 standing in a neutral posture in control condition. (PDF)

S3 Fig Bar graph showing proportion of pups that followed and did not follow pointing cues over the range of 4^th^ week– 8^th^ weeks. No significant variation was found for point-following behaviour of pups during the age range (Goodness of fit, p = 0.405). Black bars indicate pups that followed point and grey bars indicate pups that did not follow the pointing cue.
(PDF)

## References

[1] Clutton-Brock J. Domestication and Evolution. Origins of the dog: Domestication and early history. The Domestic Dog: Its Evolution, Behaviour and Interactions with People. 1995 pp. 7–50.

[2] Kaminski J, Riedel J, Call J, Tomasello M. Domestic goats, Capra hircus, follow gaze direction and use social cues in an object choice task. Anim Behav. 2005;69: 11–18.

[3] Nawroth C, Ebersbach M, von Borell E. Juvenile domestic pigs (Sus scrofa domestica) use human-given cues in an object choice task. Anim Cogn. 2014;17: 701–713. doi: 10.1007/s10071-013-0702-3 24197275 10.1007/s10071-013-0702-3

[4] Hernádi A, Kis A, Turcsán B, Topál J. Man’s Underground Best Friend: Domestic Ferrets Unlike the Wild Forms, Show Evidence of Dog-Like Social-Cognitive Skills. Chaline N, editor. PLoS One. 2012;7: e43267 doi: 10.1371/journal.pone.0043267 22905244 10.1371/journal.pone.0043267 PMC3419687

[5] Maros K, Gácsi M, Miklósi Á. Comprehension of human pointing gestures in horses (Equus caballus). Anim Cogn. 2008;11: 457–466. doi: 10.1007/s10071-008-0136-5 18247069 10.1007/s10071-008-0136-5

[6] Miklósi Á, Pongrácz P, Lakatos G, Topál J, Csányi V. A Comparative Study of the Use of Visual Communicative Signals in Interactions Between Dogs (Canis familiaris) and Humans and Cats (Felis catus) and Humans. J Comp Psychol. 2005;119: 179–186. doi: 10.1037/0735-7036.119.2.179 15982161 10.1037/0735-7036.119.2.179

[7] Marshall-Pescini S, Kaminski J. The Social Dog. The Social Dog. Elsevier; 2014 pp. 3–33.

[8] Serpell JA. Evidence for an association between pet behavior and owner attachment levels. Appl Anim Behav Sci. 1996;47: 49–60.

[9] Gácsi M, Maros K, Sernkvist S, Faragó T, Miklósi Á. Human Analogue Safe Haven Effect of the Owner: Behavioural and Heart Rate Response to Stressful Social Stimuli in Dogs. Kalueff A V., editor. PLoS One. 2013;8: e58475 doi: 10.1371/journal.pone.0058475 23469283 10.1371/journal.pone.0058475 PMC3587610

[10] Deldalle S, Gaunet F. Effects of 2 training methods on stress-related behaviors of the dog (Canis familiaris) and on the dog–owner relationship. J Vet Behav Clin Appl Res. 2014;9: 58–65.

[11] Horváth Z, Dóka A, Miklósi Á. Affiliative and disciplinary behavior of human handlers during play with their dog affects cortisol concentrations in opposite directions. Horm Behav. 2008;54: 107–114. doi: 10.1016/j.yhbeh.2008.02.002 18353328 10.1016/j.yhbeh.2008.02.002

[12] Hare B, Brown M, Williamson C, Tomasello M. The domestication of social cognition in dogs. Science. 2002;298: 1634–6. doi: 10.1126/science.1072702 12446914 10.1126/science.1072702

[13] Miklösi Á, Polgárdi R, Topál J, Csányi V. Use of experimenter-given cues in dogs. Anim Cogn. 1998;1: 113–121. doi: 10.1007/s100710050016 24399275 10.1007/s100710050016

[14] Soproni K, Miklósi A, Topál J, Csányi V. Comprehension of human communicative signs in pet dogs (Canis familiaris). J Comp Psychol. 2001;115: 122–126. 11459158 10.1037/0735-7036.115.2.122

[15] Miklósi Á, Soproni K. A comparative analysis of animals’ understanding of the human pointing gesture. Anim Cogn. 2006;9: 81–93. doi: 10.1007/s10071-005-0008-1 16235075 10.1007/s10071-005-0008-1

[16] Udell MAR, Giglio RF, Wynne CDL. Domestic dogs (Canis familiaris) use human gestures but not nonhuman tokens to find hidden food. J Comp Psychol. 2008;122: 84–93. doi: 10.1037/0735-7036.122.1.84 18298285 10.1037/0735-7036.122.1.84

[17] Soproni K, Miklósi Á, Topál J, Csányi V. Dogs’ (Canis familaris) responsiveness to human pointing gestures. J Comp Psychol. 2002;116: 27–34. 11926681 10.1037/0735-7036.116.1.27

[18] Gácsi M, Gyoöri B, Virányi Z, Kubinyi E, Range F, Belényi B, et al Explaining Dog Wolf Differences in Utilizing Human Pointing Gestures: Selection for Synergistic Shifts in the Development of Some Social Skills. Allen C, editor. PLoS One. 2009;4: e6584 doi: 10.1371/journal.pone.0006584 19714197 10.1371/journal.pone.0006584 PMC2719091

[19] Hare B, Tomasello M. Human-like social skills in dogs? Trends Cogn Sci. 2005;9: 439–444. doi: 10.1016/j.tics.2005.07.003 16061417 10.1016/j.tics.2005.07.003

[20] Dorey NR, Udell MAR, Wynne CDL. When do domestic dogs, Canis familiaris, start to understand human pointing? The role of ontogeny in the development of interspecies communication. Anim Behav. 2010;79: 37–41.

[21] Udell MAR. When dogs look back: inhibition of independent problem-solving behaviour in domestic dogs (Canis lupus familiaris) compared with wolves (Canis lupus). Biol Lett. 2015;11: 20150489 doi: 10.1098/rsbl.2015.0489 26382070 10.1098/rsbl.2015.0489 PMC4614426

[22] Miklósi Á, Topál J, Csányi V. Comparative social cognition: what can dogs teach us? Anim Behav. 2004;67: 995–1004.

[23] Reid PJ. Adapting to the human world: Dogs’ responsiveness to our social cues. Behav Processes. 2009;80: 325–333. doi: 10.1016/j.beproc.2008.11.002 19056474 10.1016/j.beproc.2008.11.002

[24] Boitani L, Ciucci P. Comparative social ecology of feral dogs and wolves. Ethology, Ecol Evol. 1995;7: 49–72.

[25] Hughes J, Macdonald DW. A review of the interactions between free-roaming domestic dogs and wildlife. Biol Conserv. 2013;157: 341–351.

[26] Lord K, Feinstein M, Smith B, Coppinger R. Variation in reproductive traits of members of the genus Canis with special attention to the domestic dog (Canis familiaris). Behavioural Processes. 2013 pp. 131–142. 10.1016/j.beproc.2012.10.009 23124015

[27] Majumder S Sen, Bhadra A, Ghosh A, Mitra S, Bhattacharjee D, Chatterjee J, et al To be or not to be social: Foraging associations of free-ranging dogs in an urban ecosystem. Acta Ethol. 2014;17: 1–8.

[28] Bhadra A, Bhattacharjee D, Paul M, Singh A, Gade PR, Shrestha P, et al The Meat of the Matter: A thumb rule for scavenging dogs? Ethol Ecol Evol. 2015;9370.

[29] Shannon LM, Boyko RH, Castelhano M, Corey E, Hayward JJ, Mclean C, et al Genetic structure in village dogs reveals a Central Asian domestication origin. Proc Natl Acad Sci. 2015;112: 13639–13644. doi: 10.1073/pnas.1516215112 26483491 10.1073/pnas.1516215112 PMC4640804

[30] Akey JM, Ruhe AL, Akey DT, Wong AK, Connelly CF, Madeoy J, et al Tracking footprints of artificial selection in the dog genome. Proc Natl Acad Sci. 2010;107: 1160–1165. doi: 10.1073/pnas.0909918107 20080661 10.1073/pnas.0909918107 PMC2824266

[31] Thapar R. A history of India. Penguin UK; 1990.

[32] Vanak AT, Gompper ME. Dietary Niche Separation Between Sympatric Free-Ranging Domestic Dogs and Indian Foxes in Central India. J Mammal. 2009;90: 1058–1065.

[33] Paul M, Sen Majumder S, Sau S, Nandi AK, Bhadra A. High early life mortality in free-ranging dogs is largely influenced by humans. Sci Rep. 2016;6: 19641 doi: 10.1038/srep19641 26804633 10.1038/srep19641 PMC4726281

[34] Fekadu M. Rabies in Ethiopia. Am J Epidemiol. 1982;115: 266–273. 7058785 10.1093/oxfordjournals.aje.a113298

[35] Sillero-Zubiri C, King AA, Macdonald DW. RABIES AND MORTALITY IN ETHIOPIAN WOLVES (CANIS SIMENSIS). J Wildl Dis. 1996;32: 80–86. doi: 10.7589/0090-3558-32.1.80 8627941 10.7589/0090-3558-32.1.80

[36] Butler JR., du Toit J., Bingham J. Free-ranging domestic dogs (Canis familiaris) as predators and prey in rural Zimbabwe: threats of competition and disease to large wild carnivores. Biol Conserv. 2004;115: 369–378.

[37] Bensky MK, Gosling SD, Sinn DL. The world from a dog’s point of view: A review and synthesis of dog cognition research. Adv Study Behav. 2013;45: 209–406.

[38] Duranton C, Gaunet F. Effects of shelter housing on dogs’ sensitivity to human social cues. J Vet Behav Clin Appl Res. 2016;14: 20–27.

[39] Miklósi A, Polgárdi R, Topál J, Csányi V. Intentional behaviour in dog-human communication: an experimental analysis of “showing” behaviour in the dog. Anim Cogn. 2000;3: 159–166.

[40] Marshall-Pescini S, Colombo E, Passalacqua C, Merola I, Prato-Previde E. Gaze alternation in dogs and toddlers in an unsolvable task: evidence of an audience effect. Anim Cogn. 2013;16: 933–943. doi: 10.1007/s10071-013-0627-x 23543361 10.1007/s10071-013-0627-x

[41] Szetei V, Miklósi Á, Topál J, Csányi V. When dogs seem to lose their nose: an investigation on the use of visual and olfactory cues in communicative context between dog and owner. Appl Anim Behav Sci. 2003;83: 141–152.

[42] Prato-Previde E, Marshall-Pescini S, Valsecchi P. Is your choice my choice? The owners’ effect on pet dogs’ (Canis lupus familiaris) performance in a food choice task. Anim Cogn. 2007;11: 167–174. doi: 10.1007/s10071-007-0102-7 17641921 10.1007/s10071-007-0102-7

[43] Takaoka A, Maeda T, Hori Y, Fujita K. Do dogs follow behavioral cues from an unreliable human? Anim Cogn. 2014;18: 475–483. doi: 10.1007/s10071-014-0816-2 25348065 10.1007/s10071-014-0816-2

[44] Appelle S. Perception and discrimination as a function of stimulus orientation: the “oblique effect” in man and animals. Psychol Bull. 1972;78: 266–278. 4562947 10.1037/h0033117

[45] Landis JR, Koch GG. The Measurement of Observer Agreement for Categorical Data. Biometrics. 1977;33: 159 843571

[46] R Development Core Team. R: A language and environment for statistical computing. R Foundation for Statistical Computing, Vienna, Austria URL http://www.R-project.org/. R Foundation for Statistical Computing, Vienna, Austria. 2015.

[47] Hare B, Tomasello M. Domestic dogs (Canis familiaris) use human and conspecific social cues to locate hidden food. J Comp Psychol. 1999;113: 173–177.

[48] Riedel J, Schumann K, Kaminski J, Call J, Tomasello M. The early ontogeny of human-dog communication. Anim Behav. 2008;75: 1003–1014.

[49] Gácsi M, Kara E, Belényi B, Topál J, Miklósi Á. The effect of development and individual differences in pointing comprehension of dogs. Anim Cogn. 2009;12: 471–479. doi: 10.1007/s10071-008-0208-6 19130102 10.1007/s10071-008-0208-6

[50] Paul M, Bhadra A. Selfish Pups: Weaning Conflict and Milk Theft in Free-Ranging Dogs. Weary D, editor. PLoS One. 2017;12: e0170590 doi: 10.1371/journal.pone.0170590 28178276 10.1371/journal.pone.0170590 PMC5298236

[51] Paul M, Majumder S Sen, Nandi AK, Bhadra A. Selfish mothers indeed! Resource-dependent conflict over extended parental care in free-ranging dogs. R Soc Open Sci. 2015;2: 150580 doi: 10.1098/rsos.150580 27019741 10.1098/rsos.150580 PMC4807463

[52] Paul M, Majumder S Sen, Bhadra A. Selfish mothers? An empirical test of parent-offspring conflict over extended parental care. Behav Processes. 2014;103: 17–22. doi: 10.1016/j.beproc.2013.10.006 24216083 10.1016/j.beproc.2013.10.006

[53] Zaine I, Domeniconi C, Wynne CDL. The ontogeny of human point following in dogs: When younger dogs outperform older. Behav Processes. 2015;119: 76–85. doi: 10.1016/j.beproc.2015.07.004 26192336 10.1016/j.beproc.2015.07.004

[54] Sen Majumder S, Paul M, Sau S, Bhadra A. Denning habits of free-ranging dogs reveal preference for human proximity. Sci Rep. 2016;6: 32014 doi: 10.1038/srep32014 27535214 10.1038/srep32014 PMC4989282

[55] Freedman DG, King JA, Elliot O. Critical Period in the Social Development of Dogs. Science (80-). 1961;133: 1016–1017. 13701603 10.1126/science.133.3457.1016

[56] Lord K. A Comparison of the Sensory Development of Wolves (Canis lupus lupus) and Dogs (Canis lupus familiaris). Zeh D, editor. Ethology. 2013;119: 110–120.

[57] Petter M, Musolino E, Roberts WA, Cole M. Can dogs (Canis familiaris) detect human deception? Behav Processes. 2009;82: 109–118. doi: 10.1016/j.beproc.2009.07.002 19686949 10.1016/j.beproc.2009.07.002

[58] Bhadra A, Bhadra A. Preference for meat is not innate in dogs. J Ethol. 2014;32: 15–22.

[59] Albiach-Serrano A, Bräuer J, Cacchione T, Zickert N, Amici F. The effect of domestication and ontogeny in swine cognition (Sus scrofa scrofa and S. s. domestica). Appl Anim Behav Sci. 2012;141: 25–35.

[60] Larson G, Dobney K, Albarella U, Fang M, Matisoo-Smith E, Robins J, et al Worldwide phylogeography of wild boar reveals multiple centers of pig domestication. Science. 2005;307: 1618–21. doi: 10.1126/science.1106927 15761152 10.1126/science.1106927

[61] Miklósi Á, Kubinyi E, Topál J, Gácsi M, Virányi Z, Csányi V. A Simple Reason for a Big Difference. Curr Biol. 2003;13: 763–766. 12725735 10.1016/s0960-9822(03)00263-x

