# Supplementary material for: Free-ranging dogs show age related plasticity in their ability to follow human pointing
Source: PLoS One. 2017 Jul 17;12(7):e0180643. doi: 10.1371/journal.pone.0180643 (PMC5513426; doi:10.1371/journal.pone.0180643)
Supplement: S1 Table — (PDF) [file pone.0180643.s001.pdf]

Free-ranging dogs show age related plasticity in their ability to follow human pointing

Debottam Bhattacharjee[1], Nikhil Dev N[1,2], Shreya Gupta[1,3], Shubhra Sau[1], Rohan Sarkar[1], Arpita Biswas[1], Arunita Banerjee[1], Daisy Babu[1], Diksha Mehta[1,4] and Anindita Bhadra[1*]

Affiliations:

[1] Department of Biological Sciences, Indian Institute of Science Education and Research Kolkata, Nadia, West Bengal, India

[2] Indian Institute of Science Education and Research Thiruvannathapuram, Kerala, India

[3] Indian Institute of Science Education and Research Bhopal, Madhya Pradesh, India

[4] Shivaji College, University of Delhi, Delhi, India

[*]Corresponding author

E-mail: abhadra@iiserkol.ac.in (AB)

S1 Table. Sample sizes used for the three age categories in the experiment for test and control conditions

	No. of pups	No. of juveniles	No. of adults
**Test condition**	36	30	42
**Control condition**	32	28	41
