# Supplementary material for: Free-ranging dogs show age related plasticity in their ability to follow human pointing
Source: PLoS One. 2017 Jul 17;12(7):e0180643. doi: 10.1371/journal.pone.0180643 (PMC5513426; doi:10.1371/journal.pone.0180643)
Supplement: S1 Text — (PDF) [file pone.0180643.s003.pdf]

**Free-ranging dogs show age related plasticity in their ability to follow human pointing**

Debottam Bhattacharjee[1], Nikhil Dev N[1,2], Shreya Gupta[1,3], Shubhra Sau[1], Rohan Sarkar[1], Arpita Biswas[1], Arunita Banerjee[1], Daisy Babu[1], Diksha Mehta[1,4] and Anindita Bhadra[1*]

Affiliations:

[1] Department of Biological Sciences, Indian Institute of Science Education and Research Kolkata, Nadia, West Bengal, India

[2] Indian Institute of Science Education and Research Thiruvannathapuram, Kerala, India

[3] Indian Institute of Science Education and Research Bhopal, Madhya Pradesh, India

[4] Shivaji College, University of Delhi, Delhi, India

[*]Corresponding author

E-mail: abhadra@iiserkol.ac.in (AB)

## S1 Text. Why kneel down posture was not used?

We have done the experiment with kneel down stance and the results are following – 11 adult dogs tested out of which only 3 individuals approached and 8 individuals did not approach or leave the place of study (Goodness of fit $\chi^2 = 21.160$, df = 1, $p < 0.001$). Thus we modified the protocol to bending over stance over kneel down for the experiment.
