# Supplementary material for: Free-ranging dogs show age related plasticity in their ability to follow human pointing
Source: PLoS One. 2017 Jul 17;12(7):e0180643. doi: 10.1371/journal.pone.0180643 (PMC5513426; doi:10.1371/journal.pone.0180643)
Supplement: S2 Text — (PDF) [file pone.0180643.s004.pdf]

Free-ranging dogs show age related plasticity in their ability to follow human pointing

Debottam Bhattacharjee[1], Nikhil Dev N[1,2], Shreya Gupta[1,3], Shubhra Sau[1], Rohan Sarkar[1], Arpita Biswas[1], Arunita Banerjee[1], Daisy Babu[1], Diksha Mehta[1,4] and Anindita Bhadra[1*]

Affiliations:

[1] Department of Biological Sciences, Indian Institute of Science Education and Research Kolkata, Nadia, West Bengal, India

[2] Indian Institute of Science Education and Research Thiruvannathapuram, Kerala, India

[3] Indian Institute of Science Education and Research Bhopal, Madhya Pradesh, India

[4] Shivaji College, University of Delhi, Delhi, India

[*]Corresponding author

E-mail: abhadra@iiserkol.ac.in (AB)

Cox Proportional Hazards test:

```
coxph(formula = Surv(dat$time, dat$status) ~ groupmod)

  n= 108, number of events= 72

                    coef exp(coef) se(coef)      z Pr(>|z|)
groupmodjuvenile -0.5602    0.5711   0.3359 -1.668  0.09535 .
groupmodpup       0.7356    2.0868   0.2632  2.795  0.00519 **
---
Signif. codes:  0 ‘***’ 0.001 ‘**’ 0.01 ‘*’ 0.05 ‘.’ 0.1 ‘ ’ 1

                 exp(coef) exp(-coef) lower .95 upper .95
groupmodjuvenile    0.5711     1.7510    0.2957     1.103
groupmodpup         2.0868     0.4792    1.2459     3.495

Concordance= 0.647  (se = 0.039 )
Rsquare= 0.148   (max possible= 0.996 )
Likelihood ratio test= 17.26  on 2 df,   p=0.000179
Wald test            = 17.04  on 2 df,   p=0.0001992
Score (logrank) test = 18.61  on 2 df,   p=9.092e-05
```

Proportional Hazard’s assumption:

```
> cxp.assum <- cox.zph(cxp)
> cxp.assum
                    rho chisq     p
groupmodjuvenile -0.109 0.864 0.353
groupmodpup      -0.144 1.427 0.232
GLOBAL               NA 1.670 0.434
```

Log Rank test:

```
> log_rank <- survdiff(Surv(dat$time, dat$status) ~ groupmod, rho = 0)
> log_rank
Call:
survdiff(formula = Surv(dat$time, dat$status) ~ groupmod, rho = 0)

                   N Observed Expected (O-E)^2/E (O-E)^2/V
groupmod=adult    42       28     30.3     0.175     0.346
groupmod=juvenile 30       13     24.1     5.128     8.876
groupmod=pup      36       31     17.6    10.255    15.943
```
