# Supplementary material for: Free-ranging dogs show age related plasticity in their ability to follow human pointing
Source: PLoS One. 2017 Jul 17;12(7):e0180643. doi: 10.1371/journal.pone.0180643 (PMC5513426; doi:10.1371/journal.pone.0180643)
Supplement: S3 Text — (PDF) [file pone.0180643.s005.pdf]

Free-ranging dogs show age related plasticity in their ability to follow human pointing

Debottam Bhattacharjee[1], Nikhil Dev N[1,2], Shreya Gupta[1,3], Shubhra Sau[1], Rohan Sarkar[1], Arpita Biswas[1], Arunita Banerjee[1], Daisy Babu[1], Diksha Mehta[1,4] and Anindita Bhadra[1*]

Affiliations:

[1] Department of Biological Sciences, Indian Institute of Science Education and Research Kolkata, Nadia, West Bengal, India

[2] Indian Institute of Science Education and Research Thiruvannathapuram, Kerala, India

[3] Indian Institute of Science Education and Research Bhopal, Madhya Pradesh, India

[4] Shivaji College, University of Delhi, Delhi, India

[*]Corresponding author

E-mail: abhadra@iiserkol.ac.in (AB)

S3 Text. Effect of frequency of gaze alternation on pup's point-following behaviour

We calculated generalized linear models (GLM) to check the effect of duration and frequency of gaze alternation behaviour on the pointing followed and not followed behaviour with a binomial distribution. We selected model 3 based on the lowest AIC value. Fixed effects and the response variables have been incorporated in the models. ANOVA test between the models did not give any significant result. Following are the models -

Model 1:

```
lrfit115 <- glm(pf ~ freq*dura , data=pup, family = binomial)

> summary(lrfit115)

Call:
glm(formula = pf ~ freq * dura, family = binomial, data = pup)

Deviance Residuals:
    Min       1Q   Median       3Q      Max
-2.0963   0.0000   0.4854   0.4854   1.4823

Coefficients:
            Estimate Std. Error z value Pr(>|z|)
(Intercept)    2.079      0.750   2.773  0.00556 **
freq          -4.159      4.250  -0.979  0.32780
dura           3.495    533.895   0.007  0.99478
freq:dura     -2.802    533.893  -0.005  0.99581
---
Signif. codes:  0 ‘***’ 0.001 ‘**’ 0.01 ‘*’ 0.05 ‘.’ 0.1 ‘ ’ 1

(Dispersion parameter for binomial family taken to be 1)
```

```
    Null deviance: 43.230  on 31  degrees of freedom
Residual deviance: 21.922  on 28  degrees of freedom
AIC: 29.922

Number of Fisher Scoring iterations: 20
```

Model 2:

```
> lrfit116 <- glm(pf ~ freq+dura , data=pup, family = binomial(link = "log
it"))
> summary(lrfit116)

Call:
glm(formula = pf ~ freq + dura, family = binomial(link = "logit"),
    data = pup)

Deviance Residuals:
    Min       1Q   Median       3Q      Max
-2.1230  -0.1825   0.4711   0.4711   1.3886

Coefficients:
            Estimate Std. Error z value Pr(>|z|)
(Intercept)   2.1425     0.7553   2.837  0.00456 **
freq         -2.1560     1.9468  -1.107  0.26809
dura         -0.1568     0.5553  -0.282  0.77760
---
Signif. codes:  0 ‘***’ 0.001 ‘**’ 0.01 ‘*’ 0.05 ‘.’ 0.1 ‘ ’ 1

(Dispersion parameter for binomial family taken to be 1)

    Null deviance: 43.230  on 31  degrees of freedom
Residual deviance: 22.475  on 29  degrees of freedom
AIC: 28.475
```

```
Number of Fisher Scoring iterations: 8
```

Model 3:

```
> lrfit117 <- glm(pf ~ freq , data=pup, family = binomial(link = "logit"))
> summary(lrfit117)

Call:
glm(formula = pf ~ freq, family = binomial(link = "logit"), data = pup)

Deviance Residuals:
    Min       1Q   Median       3Q      Max
-2.1457  -0.2772   0.4592   0.4592   1.4054

Coefficients:
            Estimate Std. Error z value Pr(>|z|)
(Intercept)   2.1965     0.7541   2.913  0.00358 **
freq         -2.7180     0.9017  -3.014  0.00258 **
---
Signif. codes:  0 ‘***’ 0.001 ‘**’ 0.01 ‘*’ 0.05 ‘.’ 0.1 ‘ ’ 1

(Dispersion parameter for binomial family taken to be 1)

    Null deviance: 43.230  on 31  degrees of freedom
Residual deviance: 22.629  on 30  degrees of freedom
AIC: 26.629

Number of Fisher Scoring iterations: 5

anova(lrfit117,lrfit116,lrfit115)
Analysis of Deviance Table

Model 1: pf ~ freq
Model 2: pf ~ freq + dura
```

```
Model 3: pf ~ freq * dura
  Resid. Df Resid. Dev Df Deviance
1        30     22.630
2        29     22.475  1  0.15478
3        28     21.922  1  0.55247
```
