# Supplementary material for: Free-ranging dogs show age related plasticity in their ability to follow human pointing
Source: PLoS One. 2017 Jul 17;12(7):e0180643. doi: 10.1371/journal.pone.0180643 (PMC5513426; doi:10.1371/journal.pone.0180643)

Free-ranging dogs show age related plasticity in their ability to follow human pointing

Debottam Bhattacharjee[1], Nikhil Dev N[1,2], Shreya Gupta[1,3], Shubhra Sau[1], Rohan Sarkar[1], Arpita Biswas[1], Arunita Banerjee[1], Daisy Babu[1], Diksha Mehta[1,4] and Anindita Bhadra[1*]

Affiliations:

[1] Department of Biological Sciences, Indian Institute of Science Education and Research Kolkata, Nadia, West Bengal, India

[2] Indian Institute of Science Education and Research Thiruvannathapuram, Kerala, India

[3] Indian Institute of Science Education and Research Bhopal, Madhya Pradesh, India

[4] Shivaji College, University of Delhi, Delhi, India

[*]Corresponding author

E-mail: abhadra@iiserkol.ac.in (AB)

Fig S3. Bar graph showing proportion of pups that followed and did not follow pointing cues over the range of 4th week – 8th weeks. No significant variation was found for point-

following behaviour of pups during the age range (Goodness of fit, p = 0.405). Black bars indicate pups that followed point and grey bars indicate pups that did not follow the pointing cue.

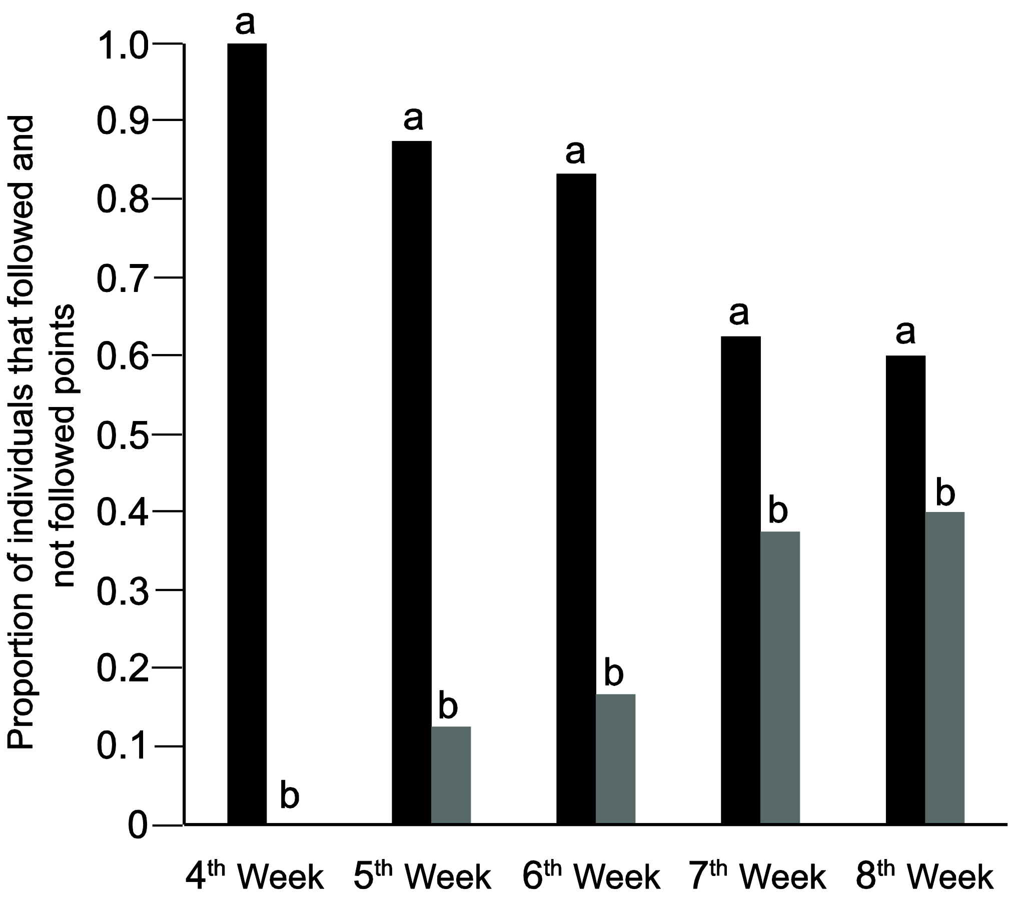

Supplement: S3 Fig — No significant variation was found for point-following behaviour of pups during the age range (Goodness of fit, p = 0.405). Black bars indicate pups that followed point and grey bars indicate pups that did not follow the pointing cue. (PDF) [file pone.0180643.s010.pdf]
